# Altered miRNA expression in pulmonary sarcoidosis

**DOI:** 10.1186/s12881-016-0266-6

**Published:** 2016-01-14

**Authors:** Justyna Kiszałkiewicz, Wojciech J. Piotrowski, Dorota Pastuszak-Lewandoska, Paweł Górski, Adam Antczak, Witold Górski, Daria Domańska-Senderowska, Monika Migdalska-Sęk, Karolina H. Czarnecka, Ewa Nawrot, Ewa Brzeziańska-Lasota

**Affiliations:** Department of Molecular Bases of Medicine, 1st Chair of Internal Diseases, Medical University of Lodz, Pomorska 251, 92-213 Lodz, Poland; Department of Pneumonology and Allergy, 1st Chair of Internal Diseases, Medical University of Lodz, Kopcińskiego 22, 90-153 Lodz, Poland; Department of General and Oncological Pulmonology, 1st Chair of Internal Diseases, Medical University of Lodz, Kopcińskiego 22, 90-153 Lodz, Poland

**Keywords:** Sarcoidosis, Molecular etiopathogenesis, Diagnostic marker, miRNA

## Abstract

**Background:**

miRNAs control important cellular functions including angiogenesis/angiostasis or fibrosis and reveal altered expression during pathological processes in the lung.

**Methods:**

The aim of the study was to investigate the expression of selected miRNAs (miR-let7f, miR-15b, miR-16, miR-20a, miR-27b, miR-128a, miR-130a, miR-192 miR-221, miR-222) in patients with pulmonary sarcoidosis (*n* = 94) and controls (n = 50). The expression was assessed by q-PCR in BALF cells and peripheral blood lymphocytes (PB lymphocytes). For statistical analysis, the Kruskal–Wallis test, Mann–Whitney U- test, Neuman–Keuls’ multiple comparison test, and Spearman’s rank correlation were used.

**Results:**

In BALF cells, significantly higher expression of miR-192 and miR-221 and lower expression of miR-15b were found in patients than controls. MiR-27b, miR-192 and miR-221 expression was significantly higher in patients without parenchymal involvement (stages I) than those at stages II-IV. Patients with acute disease demonstrated significantly higher miR-27b, miR-192 and miR-221 expression than those with insidious onset. For PB lymphocytes, patients demonstrated significantly greater miR-15b, miR-27b, miR-192, miR-221 and miR-222 expression, but lower miR-let7f and miR-130a expression, than controls. Stage I patients demonstrated significantly higher miR-16 and miR-15b expression than those in stages II-IV, and patients with the acute form demonstrated higher miR-130a and miR-15b expression. In BALF cells, miR-16 and miR-20a expression was significantly higher in patients with lung volume restriction, and miR-let7f was higher in the PB lymphocytes in patients with obturation. Several correlations were observed between the pattern of miRNA expression, lung function parameters and selected laboratory markers.

**Conclusion:**

The obtained results suggest that the studied miRNAs play a role in the pathogenesis of sarcoidosis, and that some of them might have negative prognostic value.

**Electronic supplementary material:**

The online version of this article (doi:10.1186/s12881-016-0266-6) contains supplementary material, which is available to authorized users.

## Background

Sarcoidosis is a chronic inflammatory granulomatous disorder of unknown etiology, characterized in the majority of patients by intrathoracic lymph node enlargement and parenchymal lung disease, with the possible involvement of many extrapulmonary organs.

Among many risk factors, genetic susceptibility seems to be crucial for its development in subjects exposed to unknown infectious or non-infectious environmental factors [1, 2]. This genetic predisposition to sarcoidosis is known to generally concern immunoregulatory genes [3], genes regulating various aspects of angiogenesis (e.g., cell proliferation, apoptosis, migration, morphogenesis) and angiostasis [4, 5] and genes involved in the pathogenesis of lung fibrosis [1, 6–8].

Recently, microRNAs (miRNAs) have attracted attention as being involved in many tissue and cell functions [5, 9]. Mature miRNAs act as small negative regulators of gene expression via reduction of protein translation or degradation of mRNA [10]; they are also involved in feedback interactions as *crosstalk* between many target genes.

Changes in miRNA expression levels may contribute to various lung pathologies and diseases, including cystic fibrosis, chronic obstructive pulmonary disease, asthma, idiopathic pulmonary fibrosis and lung cancer, by targeting many important signaling pathways, such as the TGF-β/SMAD signaling pathway, or by influencing HIF-1A related genes [9, 11–14].

The number of studies focused on the *crosstalk* between miRNAs and the genes involved in the pathogenesis of sarcoidosis remains limited [15–17]. Therefore, the present study evaluates the expression pattern of several miRNAs which target genes involved in proangiogenic or angiostatic functions and ECM/EMT remodeling. These are recognized as important processes in the pathogenesis of sarcoidosis, as they are involved in granulomatous formation, fibrosis, and the inhibition of the migration and proliferation of endothelial and epithelial cells [2, 4, 5, 18–21]. The study assesses the expression of miR-let7f, miR-15b, miR-16, miR-20a, miR-27b, miR-128a, miR-130a, miR-192, miR-221, and miR-222: miRNAs which target the *VEGF, TFG-B, SMADs,* and *HIF-1A* genes, as well as others under the control of hypoxia. The analyses were performed in BALF cells and peripheral blood (PB) lymphocytes of sarcoidosis patients, using the qPCR method.

## Methods

All patients signed an individual consent form. The study was approved by the Medical University of Lodz Ethics Committee (RNN/141/10/KE).

### Study group

A total of 94 patients with pulmonary sarcoidosis were recruited into the study. The patients were admitted to the Department of Pneumology and Allergy, Norbert Barlicki memorial University Hospital No. 1 in Lodz, during the years 2010-2014. The diagnosis was made based on current standards [1, 22]. For each patient, a consistent clinical and radiological picture of sarcoidosis was confirmed, with the presence of non-caseating granuloma indicated in tissue biopsy. The diagnosis was documented by EBUS-TBNA, bronchial mucosal biopsy, transbronchial peripheral lung biopsy, mediastinoscopy, or extrathoracic biopsy (skin, peripheral lymph nodes). No biopsy was obligatory only in patients with a typical clinico-radiological picture (bilateral hilar lymph nodes enlargement) and typical BAL results (increased percentage of lymphocytes with CD^+^4/CD^+^8 > 3.5). Based on their chest X-ray results, patients were classified into the following radiological subgroups: stage I (hilar lymph node enlargement without signs of parenchymal involvement), stage II (signs of parenchymal involvement in addition to hilar lymph node enlargement), stage III (parenchymal involvement without visible hilar lymph node enlargement) and IV (signs of irreversible extensive lung fibrosis). An independent comparison was performed between patients with acute disease onset (Löfgren syndrome with arthritis, erythema nodosum, elevated body temperature – with at least two symptoms present) and patients with insidious disease onset. The clinical and biological characteristics of the study group are presented Table 1.

**Table 1:** Clinical and biological characteristics of the study patients

	Stage I	Stages II-IV	Acute onset	Insidious onset
	*n* = 46	*n* = 48	*n* = 42	*n* = 51
Gender	25 F 21 M	22 F 26 M	22 F 20 M	24 F 27 M
Age	40.06	42.79	37.29	44.65
FEV_1_, % pred.	94.30 ± 14.62	87.67 ± 17.31	98.18 ± 13.59	85.10 ± 16.10
FVC, % pred.	103.54 ± 13.46	96.55 ± 117.16	107.52 ± 11.56	93.93 ± 16.21
FEV_1_/FVC	0.76 ± 0.07	0.747 ± 0.070	0.76 ± 0.07	0.74 ± 0.06
DLCOc, % pred.	-	90.73 ± 16.16	-	91.63 ± 16.69
BAL-L%	30.58 ± 18.00	29. 45 ±17.08	35.12 ± 16.62	25.79 ± 17.32
BALF CD4^+^/CD8^+^	6.84 ± 4.37	4.29 ± 3.66	7.98 ± 4.31	3.89 ± 3.18
Ca^2+^S, [mmol/l	2.44 ± 0.09	2.42 ± 0.18	2.41 ± 0.16	2.45 ± 0.13
Ca2 + U, mmol/24 hrs	4.83 ± 2.31	4.44 ± 2.66	4.24 ± 2.54	4.91 ± 2.46

Sarcoidosis patients were grouped according to the absence/presence of lung parenchymal changes on chest X-ray (stage I vs stage II-IV) and clinical phenotype (acute vs insidious onset). Abbreviations: *BAL%* broncholaveolar lavage - % of lymphocytes, *Ca* ^*2+*^ *S* serum calcium concentration, *Ca* ^*2+*^ *U* calcium in 24 hrs urine collection, *DLCOc* lung diffusion capacity for carbon monoxide corrected for hemoglobin, *FEV*_*1*_ forced expiratory volume in 1st second of expiration, *FVC* forced vital capacity. Data are presented as mean ± standard deviation

The control group consisted of healthy non-smoking persons, referred for bronchoscopy due to chronic cough or the presence of undefined changes on a chest X-ray. If the radiological signs were defined as clinically insignificant changes or artifacts, the patients underwent a thorough examination and were finally diagnosed either with idiopathic cough, or as healthy subjects. A group of 50 subjects was included for gene expression analysis in BALF cells while 20 subjects were included for gene expression analysis in PB lymphocytes.

### Bronchoscopy and bronchoalveolar lavage fluid (BALF) collection

**Fig. 1:**
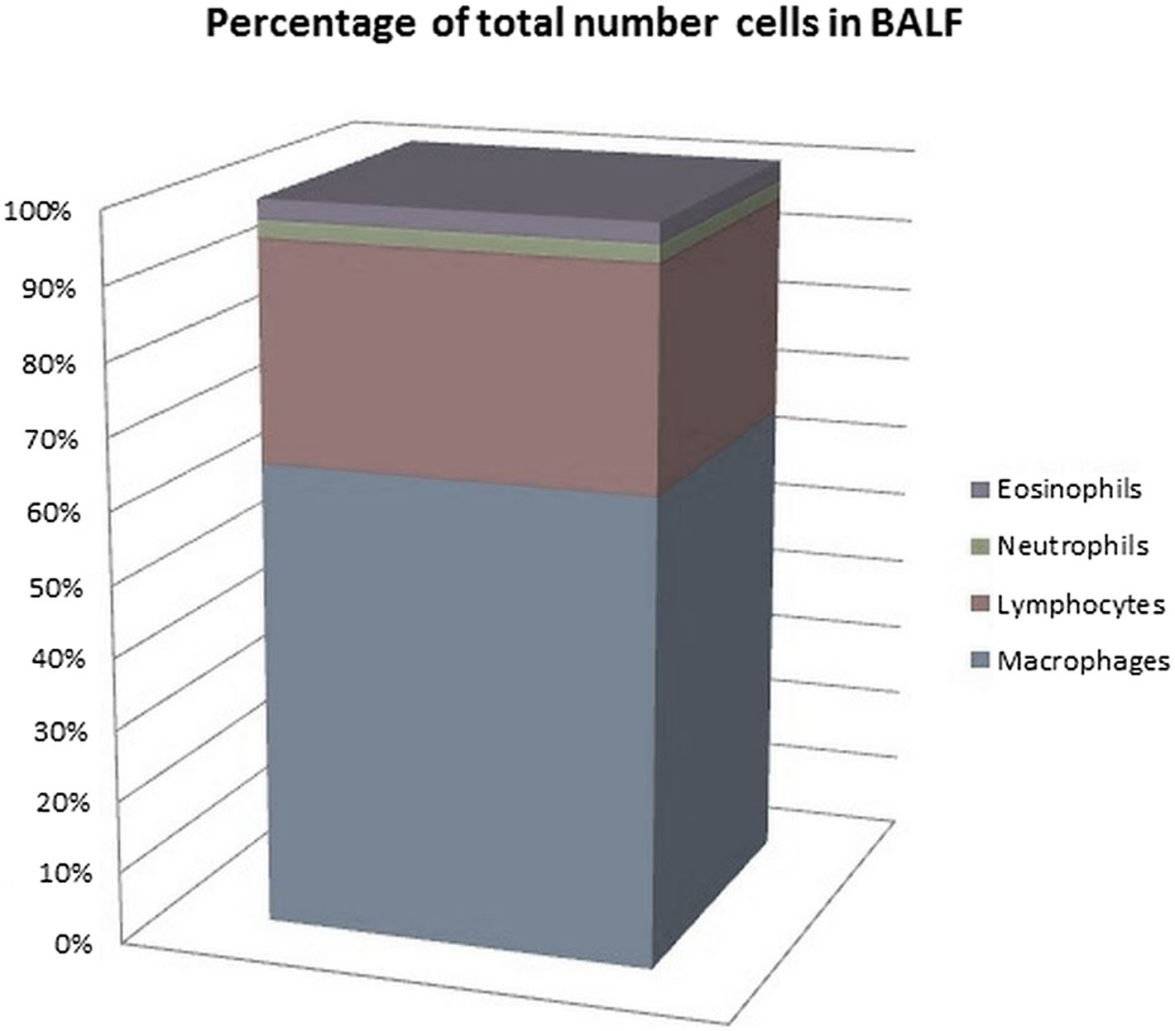
Graphs presenting the percentage of lymphocytes, neutrophils and eosinophils obtained from BALF

Bronchoscopy was performed with a flexible bronchoscope (Pentax, Tokyo, Japan) according to Polish Respiratory Society guidelines [23]. Patients optionally received midanium and atropine before the examination, and 2 % lidocaine was used as a topical anesthetic. BAL fluid (BALF) was collected from medial lobe by instillation and subsequent withdrawal of 4 x 50 ml of 0.9 % NaCl. The fluid recovery was 52.1 ± 1.2 %. The crude BALF was filtered through a gauze to clear the thick mucus and other contaminants, centrifuged, and the pellet was suspended in a phosphate buffer. The total number of non-epithelial cells (total cell count – TCC) was presented as n x 10^6^. Cytospin slides were prepared and stained by May-Grünwald-Giemsa stain. The number of macrophages, lymphocytes, neutrophils, and eosinophils was calculated under a light microscope and presented as % of TCC. After the calculations, the fluid was centrifuged (10 min at 1200 rpm) and the BALF supernatant was suspended in around 350 μl RNAlater RNA Stabilization Reagent (QIAGEN, Hilden, Germany), and frozen (-80 °C) until further RNA isolation procedures could be performed. The percentages of the numbers of macrophages, lymphocytes, neutrophilis and eosinophils obtained from the BALF are shown in Fig. 1.

### Lung function

Spirometry was performed according to the Polish Respiratory Society Guidelines [23] with a computer-based spirometer (Jaeger, Dortmund, Germany). Forced vital capacity (FVC) and forced expiratory volume in 1 s (FEV_1_) were measured, and the Tiffenau index (FEV_1_/FVC) was calculated. Lung diffusion capacity for carbon monoxide was measured in patients with lung parenchymal disease only (stage II-IV) by the single breath method, using a Lungtest 1000 SB (MES, Cracow, Poland) according to ATS/ERS standards [24]. The values were corrected for the hemoglobin concentration (DLCOc). All data (except the Tiffeneau index) was expressed as % of predicted value.

### Peripheral blood sample collection

Blood was collected into 5 ml tubes containing EDTA. For lymphocyte separation, Histopaque-1077 density gradient cell separation medium (Sigma-Aldrich, Poznan, Poland) was used according to the manufacturers protocol. The supernatant was discarded and cells were resuspended in 350 μl RNAlater RNA Stabilization Reagent before being frozen at -80 °C.

### miRNA expression analysis

RNA isolation was performed using a mirVana™ miRNA Isolation Kit (Life Technologies, Carlsbad, CA, USA), according to the manufacturer’s protocol. The quality and quantity of isolated RNA was assessed spectrophotometrically (Eppendorf BioPhotometrTM Plus, Eppendorf, Hamburg, Germany). The purity of total RNA was determined using RNA Nano Chips LabChipplates (ratio of 16S to 18S fraction) and miRNA was evaluated by automated electrophoresis using RNA Pico Chips LabChipplates in an Agilent 2100 Bioanalyzer (Agilent Technologies, Santa Clara, CA).

cDNA was transcribed from 100 ng of total RNA using a TaqMan® MicroRNA Reverse Transcription Kit (Applied Biosystems, Carlsbad, CA) in a total volume of 12 μl according to the manufacturer’s protocol.

The relative expression of miRNAs was assessed by qPCR reactions using Custom TaqMan® Array MicroRNA Cards (Applied Biosystems, Carlsbad, CA) according to the manufacturer’s protocol. The following microRNA probes were used for the study: hsa-let-7f (UGAGGUAGUAGAUUGUAUAGUU), hsa-miR-15b (UAGCAGCACA UCAUGGUUUACA), hsa-miR-16 (UAGCAGCACGUAAAUAUUGGCG), hsa-miR-20a (ACUGCAUUAUGAGCACUUAAAG), hsa-miR-27b (AGAGCUUAGCUGAUUGGUGA AC), hsa-miR-128 (UCACAGUGAACCGGUCUCUUU), hsa-miR-130a (CAGUGCAAUG UUAAAAGGGCAU), hsa-miR-192 (CUGCCAAUUCCAUAGGUCACAG), hsa-miR-221 (ACCUGGCAUACAAUGUAGAUUU), hsa-miR-22(CUCAGUAGCCAGUGUAGAUCCU).

The PCR mixture contained 0.90 μl RT Product, 56.25 μl TaqMan® Universal Master Mix II, No AmpErase® UNG (2X) and 55.35 μl Nuclease-free water (Applied Biosystems, Carlsbad, CA). TaqMan Array card was centrifuged according to manufactured protocol and placed in the Applied Biosystems 7900HT Fast Real-Time PCR System (Applied Biosystems, Carlsbad, CA) according to manufactured protocol. The expression levels (RQ values) of the studied miRNA were calculated using the delta delta CT method, with the adjustment to the level of 001973 U6 snRNA expression (endogenous control) and to the expression of the calibrator (Human Lung Total RNA Ambion®), for which RQ value was equal to 1.

### Statistical analysis

The Kruskal–Wallis test, Mann–Whitney *U*-test, Neuman–Keuls’ multiple comparison test, and Spearman’s rank correlation were used to assess the correlations between relative miRNA expression and sarcoidosis in the patient groups classified based on radiological examination (stages I vs. II-IV), acute vs. chronic disease onset, spirometric parameters, DLCO, serum Ca^2+^ concentration, Ca^2+^ loss in urine over 24 h, the percentage of lymphocytes in BAL (BALF%), the phenotype of BAL immune cells (CD4^+^/CD8^+^), age and sex of patients. *P* = 0.05 was regarded as the level of statistical significance (StatSoft, Cracow, Poland).

## Results

### Relative expression levels of the studied miRNAs in sarcoidosis patients vs. controls

#### BALF cells

**Fig. 2:**
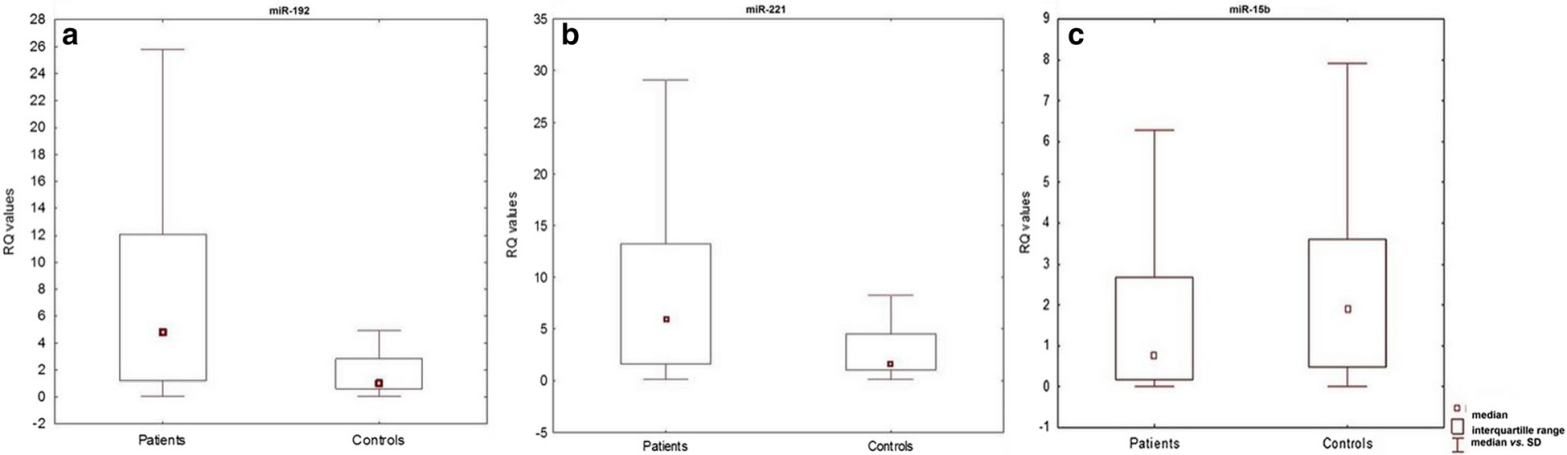
Box and whisker plots, presenting **a**) miR-192 **b**) miR-221 **c**) miR-15b expression mean values in BALF cells of patients and controls (*P = 0.00004*, Mann-Whitney U- test)

The Mann-Whitney *U*-test revealed significantly greater miRNA expression in sarcoidosis patients miR-192 (*P* = 0.00004, Mann-Whitney U- test) and for miR-221 (*P* = 0.0002, Mann-Whitney U- test) (see Fig. 2). Conversely, significantly greater miR-15b expression was noted in the control group (*P* = 0.04, Mann-Whitney U- test).

No statistically significant differences were observed between patients and controls for the remaining analyzed miRNAs (*P* > 0.05, Mann-Whitney U- test) (see Additional file 1: Figure S1).

### Peripheral blood (PB) lymphocytes

**Fig. 3:**
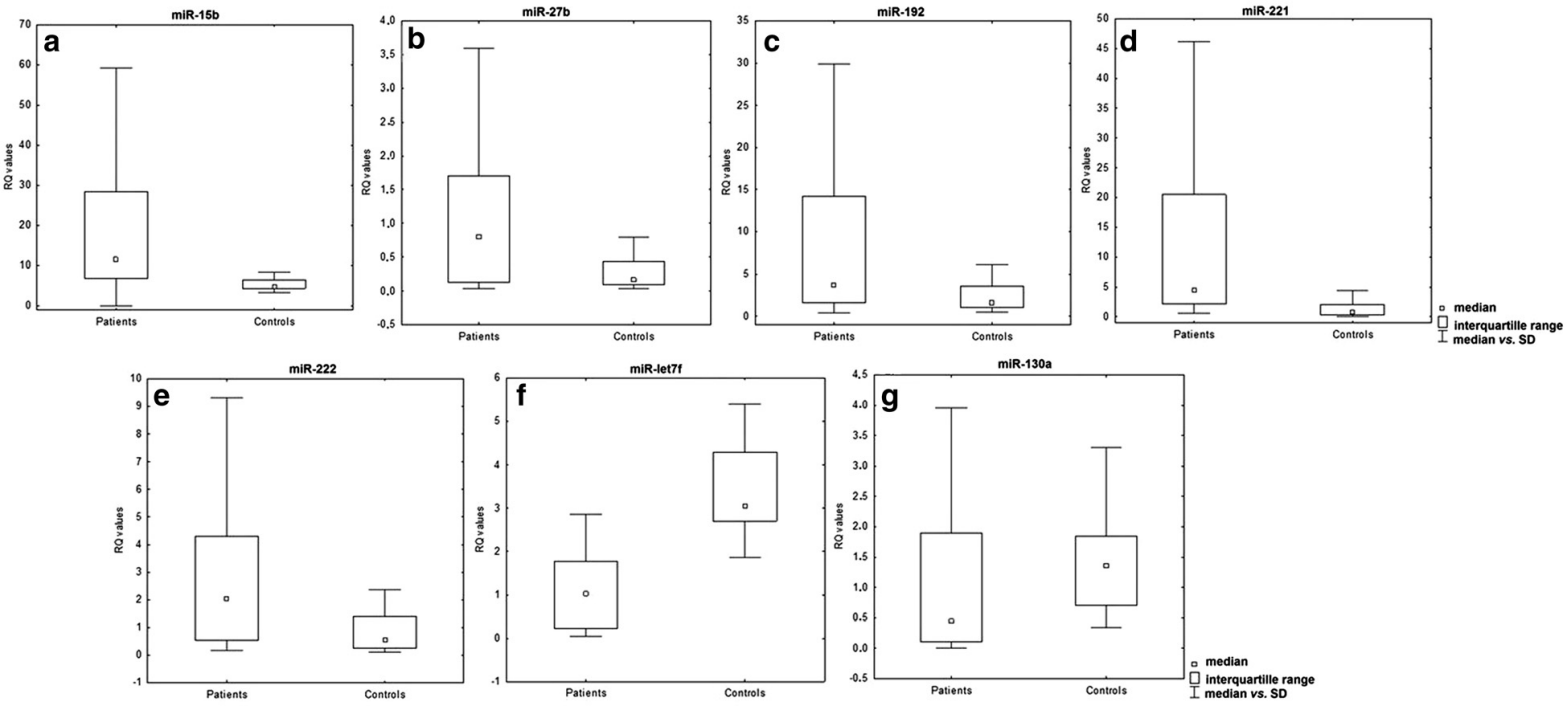
Box and whisker plots, presenting **a**) miR-15b **b**)miR-27b **c**) miR-192 **d**) miR-221 **e**) miR-222 **f**) miR-letf7 **g**) miR-130a expression (mean values) in the PB lymphocytes of patients and controls (*P = 0.0002*, Mann-Whitney U- test)

Statistically significant differences (Mann-Whitney U- test) between patients and controls were observed for miR-15b (*P* = 0.00009, Mann-Whitney U- test), miR-27b (*P* = 0.01, Mann-Whitney U- test), miR-192 (*P* = 0.005, Mann-Whitney U- test), miR-221 (*P*, Mann-Whitney U- test), and miR-222 (*P* = 0.0061, Mann-Whitney U- test) with higher miRNA expression levels in sarcoidosis patients. Statistically significant differences (Mann-Whitney U- test) were also found for miR-let7f (*P* = 0.00004, Mann-Whitney U- test), and miR-130a (*P* = 0.02, Mann-Whitney U- test), with higher miRNA expression levels in the control group (see Fig. 3).

No statistically significant differences were found between patients and controls for the rest of analyzed miRNAs (*P* > 0.05, Mann-Whitney U- test) (see Additional file 2: Figure S2).

### Relative expression of the studied miRNAs in sarcoidosis patients according to clinical and radiological classification

#### BALF cells

**Fig. 4:**
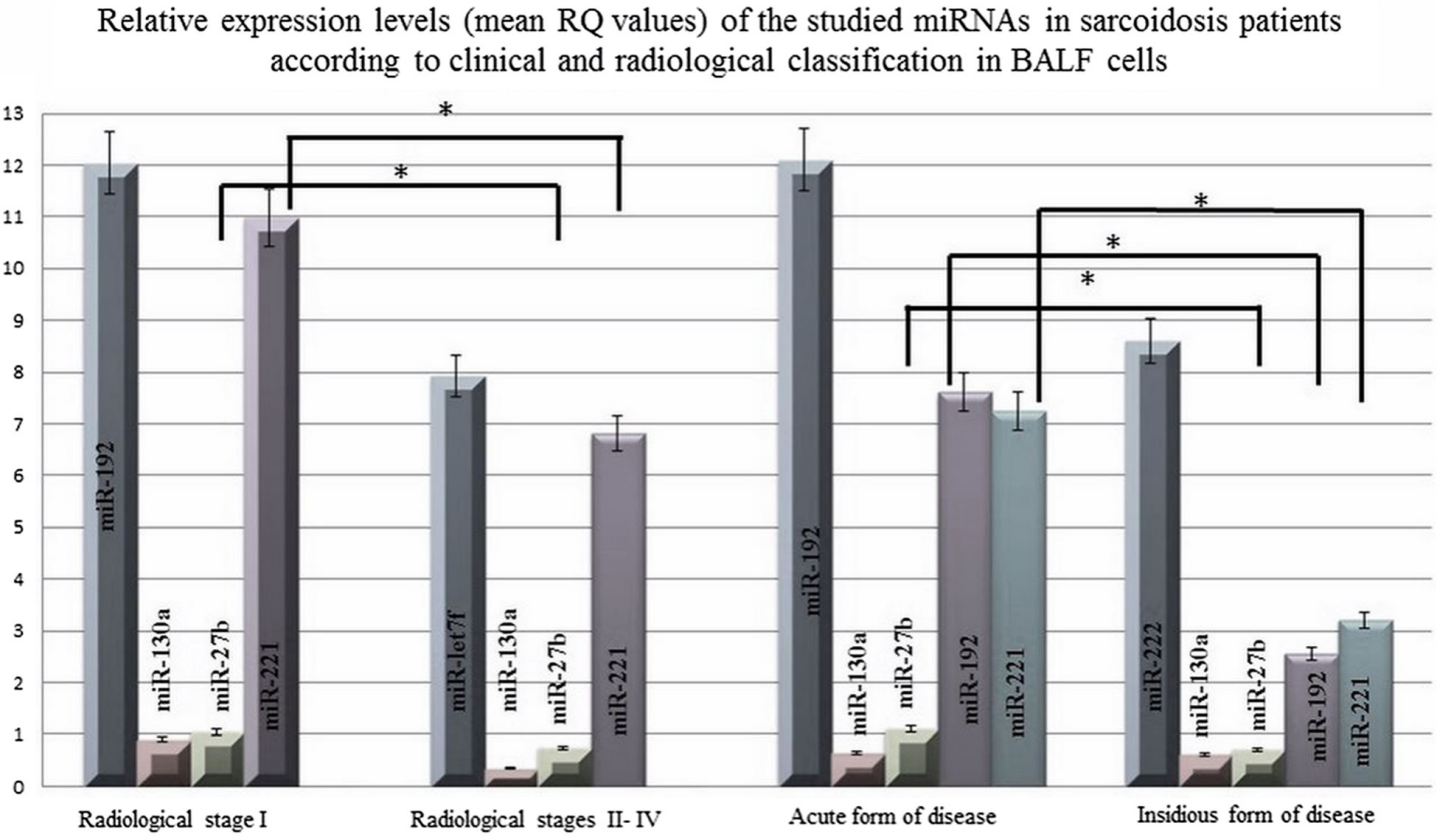
Relative expression (mean RQ values) of the studied miRNAs in sarcoidosis patients according to the clinical and radiological classification in BALF cells

In patients with radiological stage I, miR-192 demonstrated the greatest mean expression of the evaluated miRNAs (mean RQ = 12.03) and miR-130a the lowest (mean RQ = 0.907). In patients with radiological stages II-IV, the highest mean expression was associated with miR-let7f (mean RQ = 7.92) and the lowest with miR-130a (mean RQ = 0.343). In the acute form of disease, as with radiological stage I, miR-192 demonstrated the greatest mean expression (mean RQ = 12.099) and miR-130a the lowest (mean RQ = 0.637). Finally, in the insidious form of the disease, miR-222 demonstrated the highest mean expression (mean RQ = 8.600) and miR-130a the lowest (mean RQ = 0.615) (see Fig. 4).

The expression of miR-130a was decreased in the majority of studied samples (86–96 %), and the expression of miR-192 was increased in the majority of studied samples (74-91 %), regardless the of the clinical disease classification of the group.

Significantly greater miRNA expression was observed in patients without parenchymal involvement (stages I) than those with involvement (Stages II-IV) for miR-27b (*P* = 0.04), miR-192 (*P* = 0.02, Mann-Whitney U- test) and for miR-221 (*P* = 0.03, Mann-Whitney U- test) (see Additional file 3: Figure S3).

Additionally, significantly higher miRNA expression was found in patients with the acute form of disease for miR-27b (*P* = 0.002, Mann-Whitney U- test), miR-192 (*P* = 0.01, Mann-Whitney U- test), and miR-221 (*P* = 0.02, Mann-Whitney U- test) (see Additional file 4: Figure S4).

**Fig. 5:**
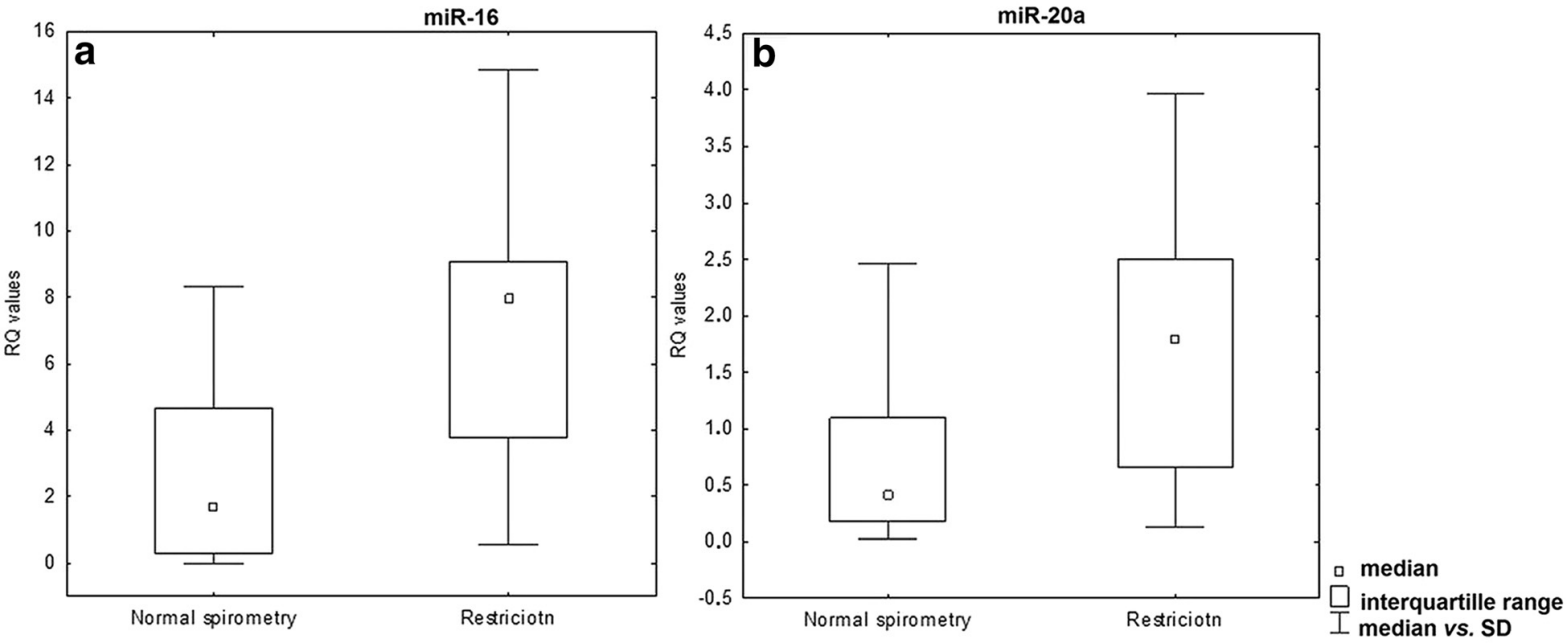
Box-and-whisker plots representing the expression of **a**) miR-16 in BALF cells (*P = 0.009*; Mann-Whitney U- test) in patients with normal spirometry and with lung volume restriction, **b**) miR-20a in BALF cells (*P = 0.04*; Mann-Whitney U- test) in patients with normal spirometry and in patients with lung volume restriction

No statistically significant differences were observed in the expression of the studied miRNAs in BALF cells for patients with normal and abnormal spirometry results (*P* > 0.05, Mann-Whitney U- test) (see Additional file 5: Figure S5). However, the expression of miRNA-16 and miRNA-20a in BALF cells was found to be significantly elevated in the subgroup of patients with a restrictive spirometric pattern (*P* = 0.009 and *P* = 0.04, respectively, Mann-Whitney U- test, see Fig. 5 and Additional file 6: Figure S6).

### PB lymphocytes

In patients with radiological stage I, miR-15b was found to have the highest mean expression of the observed miRNAs (mean RQ = 28.52) and miR-20a the lowest (mean RQ = 1.057). In patients with radiological stages II-IV, miR-221 demonstrated the highest mean expression (mean RQ = 11.5840) and miR-130a the lowest (mean RQ = 1.081).

**Fig. 6:**
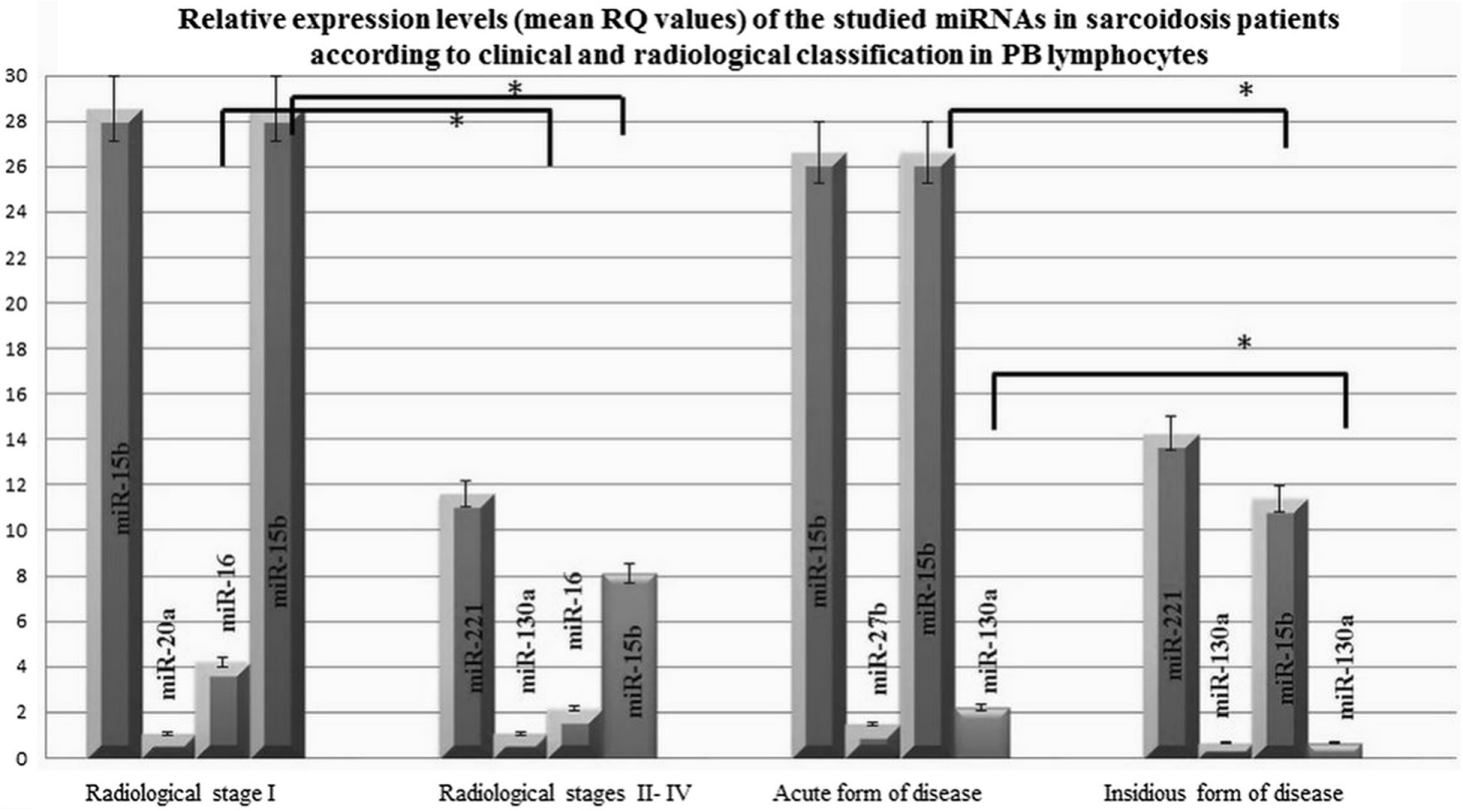
Relative expression (mean RQ values) of the studied miRNAs in sarcoidosis patients according to the clinical and radiological classification in PB lymphocytes

In the acute onset group, miR-15b was the highest (mean RQ = 26.61) and miR-27b the lowest (mean RQ = 1.478), while in the insidious onset group, miR-221 demonstrated the greatest expression (mean RQ = 14.267) and miR-130a the lowest (mean RQ =0.670) (see Fig. 6).

The expression of miR-221 was elevated in the majority of studied samples (89–100 %), while that of miR-130a was decreased (50-78 %), regardless of the clinical disease classification.

Significantly higher expression of miRNA-16 (*P* = 0.05, Mann-Whitney U- test) and miR-15b (*P* = 0.001, Mann-Whitney U- test) was observed in patients without parenchymal involvement (see Additional file 7: Figure S7), while miRNA-130a (*P =* 0.04, Mann-Whitney U – test) and miR-15b (*P* = 0.004, Mann-Whitney U- test) demonstrated higher expression in patients with the acute form of disease (see Additional file 8: Figure S8). Additionally, significantly increased expression of miRNA-let7f was found in the subgroup of patients with obturation (*P* = 0.001, Mann-Whitney U- test) (see Additional file 9: Figure S9).

### Relative expression levels of the studied miRNAs in relation to patient gender, age, selected biochemical markers, immunological phenotype and lung function test parameters

#### BALF cells

Several correlations were found between miRNA expression levels and lung function test results, as well as with selected laboratory markers in sarcoidosis patients (see Table 2). No statistically significant correlations were found between miRNA expression and gender (*P* > 0.05, Mann-Whitney *U*-test).

**Table 2:** Significant correlations found in the BALF cells and PB lymphocytes of sarcoidosis patients

BALF cells	Parameter	miRNA	Group/subgroup	Rho value	*P*-value
	AGE	miR-let7f	Parenchymal involvement	-0.44	0.001
	AGE	miR-let7f	Insidious onset	-0.34	0.01
	Ca^2+^U	miR-let7f	Acute onset	-0.45	0.04
	CD4:CD8	miR-130a	Parenchymal involvement	0.50	0.002
	FVC	miR-15b	Insidious onset	-0.33	0.02
	BALFL%	miR-192	Acute onset	-0.37	0.04
	FEV_1_/FVC	miR-16	No parenchymal involvement	-0.41	0.007
	FEV_1_/FVC	miR-16	Acute onset	-0.34	0.04
	Age	miR-16	Parenchymal involvement	-0.31	0.03
	FEV_1_	miR-20a	Acute onset	-0.36	0.03
	Ca^2+^U	miR-128a	Acute onset	-0.49	0.03
PB lymphocytes	Age	miR-15b	Entire	-0.33	0.04
	Ca^2+^U	miRlet7f	Insidious onset	0.72	0.02
	CD4:CD8	miR-128	Entire	-0.40	0.03
	Ca^2+^S	miR-27b	No parenchymal involment	-0.59	0.02
	Ca^2+^U	miR-27b	Insidious onset	0.64	0.05

Abbreviations: *BAL* bronchoalveolar lavage, *Ca* ^*2+*^ *S* serum calcium concentration, *Ca* ^*2+*^ *U* calcium in 24 hrs urine collection, *DLCOc* lung diffusion capacity for carbon monoxide corrected for hemoglobin, *FEV*_*1*_ forced expiratory volume in 1^st^ second of expiration, *FVC* forced vital capacity, *PB* peripheral blood; *P-*values of <0.05 were considered statistically significant

### PB lymphocytes

Significantly higher expression of miRNA-let7f was observed in men with chronic form of disease (*P* = 0.03, U Mann-Whitney test). Correlations between miRNA expression level and age, immunological phenotype, lung function test parameters, and selected laboratory markers in sarcoidosis patients are shown in Table 2.

### Relative expression levels of the studied miRNAs: BALF cells *vs* PB lymphocytes in sarcoidosis patients

The majority of studied BALF cell samples demonstrated higher expression of miR-let-7f, miR-16, miR-192, miR-222, but lower expression of miR-15b, miR-20a, miR-27b, miR-128, miR-130, miR-221 compared to PB lymphocytes, regardless of the clinical disease classification groups.

**Fig. 7:**
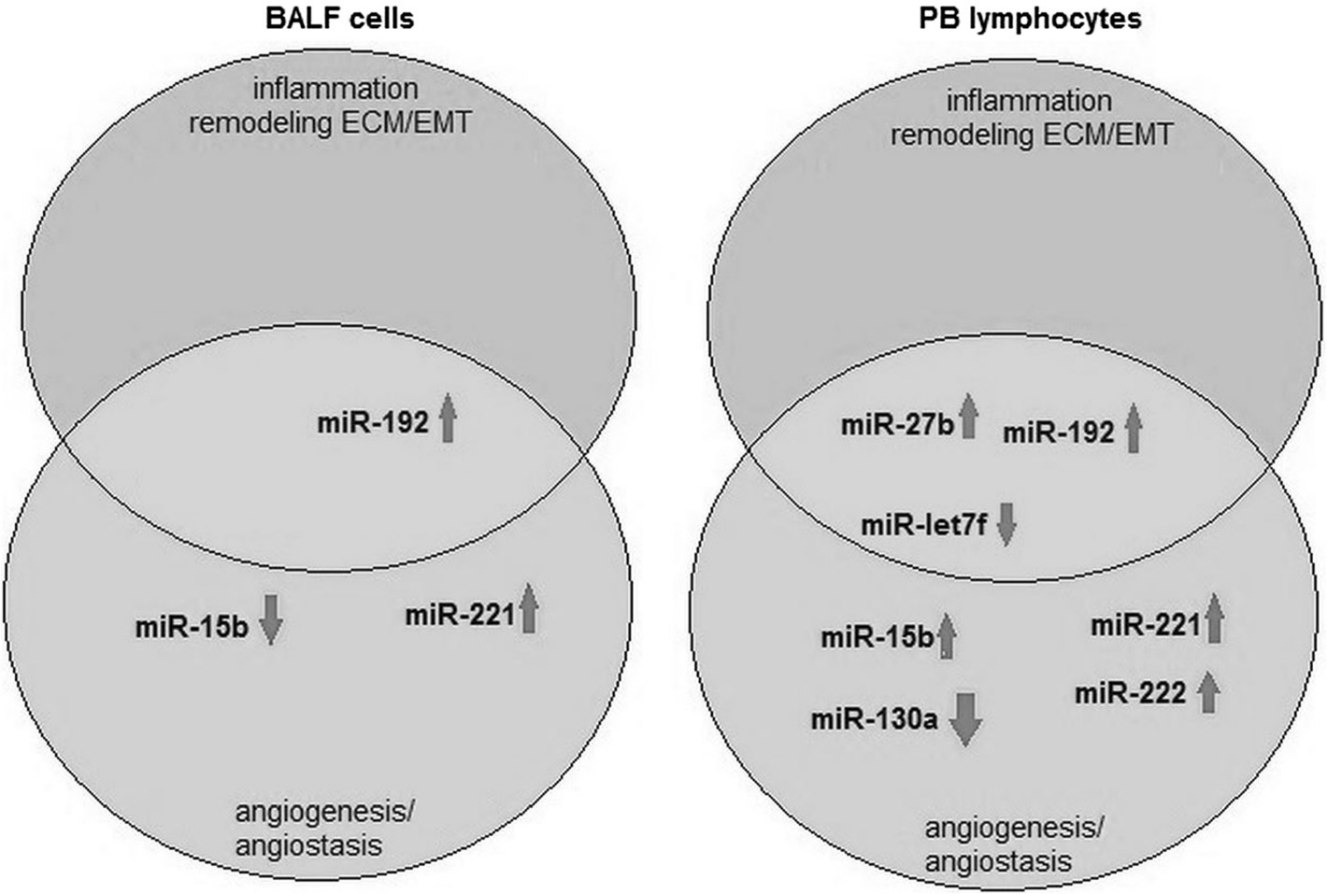
The expression pattern of miRNAs (mean RQ value) in BALF cells and PB lymphocytes in the sarcoidosis patient group

Only two of the studied miRNAs, miR-16 and miR-128a, demonstrated overlapping patterns in BALF cells and PB lymphocytes (see Fig. 7)

However, statistically significant differences between BALF cells and PB lymphocytes were only observed for three of the non-overlapping miRNAs: for miRNA-15b (*P* = 0.00001, Mann-Whitney U- test) and miRNA-130a (*P* = 0.000012, Mann-Whitney U- test), with higher expression in PB lymphocytes, and for miRNA-let7f, with higher expression levels in BALF cells (*P* = 0.000003, Mann-Whitney U- test).

## Discussion

Recent studies have found non-coding regulatory RNAs to play significant roles in the direct regulation of many genes and signaling pathways, as well as other miRNAs [5, 9, 24–26]. However, their biological role is not fully understood. Previous studies regarding miRNA interactions in the lung have mainly focused on the control of innate and adaptive lung inflammation [17, 27, 28], pulmonary fibrosis (IPF, idiopathic pulmonary fibrosis) and the regulation of extracellular matrix (ECM) remodeling [16, 29, 30]. Few studies examine miRNA expression in the course of sarcoidosis [17, 31], and the results which have been obtained are controversial. Therefore, our study offers an innovative and valuable insight into the area, not only because it addresses miRNA expression levels in the peripheral circulation, but it also attempts to determine their value in the clinical classification and monitoring of sarcoidosis patients.

Our findings are difficult to compare with those of other studies, as they use a range of biological materials to investigate the studied miRNAs. In addition, the examined miRNAs may vary with regard to their origin (free circulating miRNA or exosomal miRNA), profile and concentration. Studies on pulmonary sarcoidosis have used different classes of miRNAs and diverse biological material for miRNA isolation (BALF cells, peripheral blood mononuclear cells and serum) [17, 31]. The most recent studies, by Qazi et al [32] and Ho et al. [33], suggest that exosomes may be suitable for miRNA assessment in sarcoidosis. They note the presence of an elevated level of exosomes in BALF from sarcoidosis patients and suggest that exosomes may contribute to the inflammatory state of sarcoidosis via stimulation of cytokine production from peripheral mononuclear cells. On the other hand, exosome production is common in various cells and the mechanisms by which they control the inflammatory process are not fully understood, as well as the biological functions of miRNAs derived from the exosomes.

As miRNAs play a role in cellular communication, the most beneficial approach would be to assess their role in a particular signaling pathway which is important in sarcoidosis, irrespective of their varied origins. It should be emphasized that microarray-based studies have confirmed that alterations in miRNA expression influence the pathogenesis of sarcoidosis, as well as its relationship with the TGF-β and WNT/β-catenin signaling pathways [31]. Therefore, for the purposes of the present study, several novel miRNAs were chosen which were highly complementary to genes involved in TGF-β/Smad (regulation of fibrosis) and HIF-1A/VEGF/ING-4 (angiogenesis/angiostasis homeostasis) signaling. This choice was based on their being awarded high target scores according to the miRDB database.

The present study focuses on these two pathways because an important pathogenic component of sarcoidosis is pulmonary fibrosis, a condition associated with chronic inflammation, whose symptoms are known to be impaired wound healing and angiogenesis. Our earlier study assesses the expression of genes involved in these two pathways, i.e. the regulation of fibrosis and angiogenetic/angiostatic homeostasis, in patients with sarcoidosis [6, 34]. Our findings reveal statistically significant correlations between the expression of some of the studied miRNAs and the expression of genes which had been studied earlier [6, 34].

The present study compares the expression of selected miRNAs in PB lymphocytes and BALF cells to analyze their usefulness as specific systemic and/or local sarcoidosis markers. Although significant differences were found between patients and controls regarding the expression of several examined miRNAs in PB lymphocytes (miR-15b, miR-27b, miR-192, miR-221, miR-222, miR-130a, miR-let-7f), only three miRNAs were found to differ in BALF cells (miR-15b, miR-192, and miR-221). Our findings suggest that the above-mentioned miRNAs may be vital molecular markers for the disease, and merit further examination. It should be pointed that the biomarkers found in BALF cells appear to have high diagnostic and/or prognostic value, and may also confirm the specificity of changes in miRNA expression occurring in response to the lung microenvironment modifications which take place during sarcoidosis. We believe that of the two examined cell types, miRNA analysis may have the highest diagnostic value when performed in BALF cells. Moreover, it is interesting that the expression patterns of two studied miRNAs, miR-16 and miR-128a, overlapped in the BALF cells and PB lymphocytes of sarcoidosis patients, and their expression was higher in patients than in controls (see Fig. 5).

It is commonly known that increases in the numbers of T-cells induced by chemokines secreted by macrophages and epithelial cells, as well as shifts in the Th1/Th2 balance, play crucial roles in the course of sarcoidosis. A recent report by Barczyk et al. [35] demonstrates that CCL4 is highly expressed in T-cells (especially in CD8+, less so in CD4+) and may serve to recruit new T cells to the site of inflammation. As a prolonged state of macrophage activation and CD4+ T-lymphocyte accumulation in the presence of chronic antigenic stimulation is known to lead to inflammation in the early stage of sarcoidosis, “early sarcoid reaction” [36] and granuloma formation, an earlier study by Jazwa et al evaluated the expression of miR-15b and miR-16 as important regulators of CD4+ T cells [17]. Based on clinical and radiological classifications, significant overexpression of miR-15b and miR-16 in PB lymphocytes was observed in patients with stage I disease and in patients with the acute form of the disease. A negative correlation was also found between miR-16 expression and a lung function parameter (FEV1/FVC), and significantly higher miR-16 expression was observed in patients with lung volume restriction. The results suggest that miR-15b and miR-16 may be potentially useful prognostic markers in relation to disease progression and worse prognosis. Therefore, the influence of both miRNAs on VEGF, a target gene in angiogenesis and an inflammatory cascade regulator in sarcoidosis, seems to be pivotal in sarcoidosis pathogenesis, regarding the hypoxia-angiogenesis process and chronic inflammation in early stages of the disease, as emphasized in previous studies [37, 38]. Our previous study also confirmed the significant role played by the up-regulation of VEGF mRNA in sarcoidosis [34].

However, our results regarding miR-15b and miR-16 are not in line with those of Jazwa et al. [17], who did not report any statistically significant differences between patients and healthy controls with regard to the expression of these miRNAs.

Our present study also evaluates the expression of miR-let7f. This miRNA belongs to the class that targets TGF-β [39], a key cytokine responsible for lung fibrosis and epithelial-mesenchymal transition (EMT) [40–42]. It has been confirmed that in the lung tissue, miR-let7 influences the epithelial expression of mesenchymal markers and deposition of collagen without additional injury, which may suggest that the dysregulation of miR-let7 can lead to the overexpression of crucial pro-fibrotic factors and may be involved in the pathogenesis of pulmonary fibrosis [13, 43, 44]. Our findings reveal increased miR-let7f expression in BALF cells in patients, and a positive correlation between miR-let7f expression and Ca2 + U in the PB lymphocytes of the insidious onset group of patients. According to RTG staging (I, II-IV), the greatest mean expression of miR-let7f was observed during RTG stages II-IV. As calcium metabolism dysregulation occurs in the course of sarcoidosis and the presence of hypercalciuria and hypercalcemia in 20-50 % of patients [45], miR-let7f up-regulation may be a valuable marker in the prediction of calcium metabolism dysregulation during sarcoidosis.

To the best of our knowledge, this is the first study to examine the expression of miR-20a and miR-27b in sarcoidosis. Nevertheless, it has already been recognized that the miR-17 ~ 92 cluster, encoding six different miRNAs, is decreased in patients with IPF [46]. In the case of miR-20a and miR-27b, both are recognized as important regulators of the TGF-β/Smad pathway and fibrosis in the lung, with miR-20a additionally targeting VEGF [47]. In the present study, miR-27b expression was found to be increased in the PB lymphocytes of sarcoidosis patients, but this level was significantly lower in the advanced stages of the disease (RTG stage II-IV) than in RTG stage I. Our findings may suggest that miRNA-27b inhibition takes place during disease progression. This miRNA has been shown to play a regulatory role in pro-fibrotic changes in pulmonary cells in response to TGF-β signaling [48]. In addition, reductions in miR-27b expression were also found to be associated with TGF-β stimulation [48].

In the present study, interesting results were obtained in the case of miR 192, which showed significant up-regulation in both BALF cells and PB lymphocytes. However, its expression was found to be significantly reduced during disease progression (in stage II-IV), which is consistent with the results obtained by Hong et al. [49], who confirmed the reduction of miR 192 expression via TGF-β/Smad3 interaction in a study on chronic renal fibrosis [49].

**Fig. 8:**
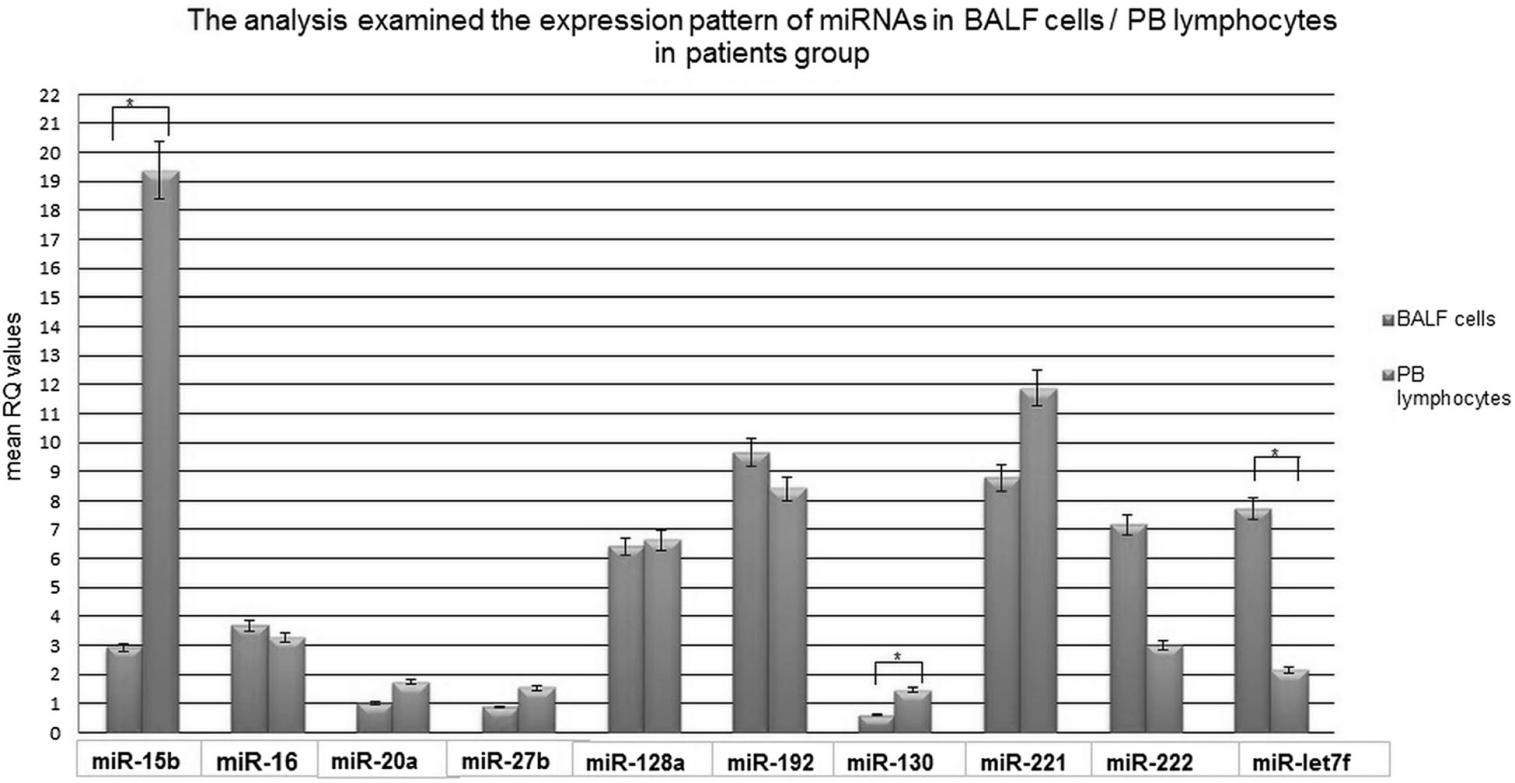
The differences in miRNA expression profiles (in BALF cells and PB lymphocytes) between controls and patients with sarcoidosis

In conclusion, the data presented in this study indicate that selected miRNA may enable patients with sarcoidosis to be distinguished from potentially healthy controls, based on the different expression profiles of the two groups (see Fig. 8).

## Conclusion

The results of this study indicate that the expression profiles of selected miRNA allow patients with sarcoidosis to be distinguished from potentially healthy controls. Moreover, it should be emphasized that some of these miRNAs may be themselves recognized as markers of disease progression, due to their elevated expression in patients with lung volume restriction or obturation. The deregulated miRNA expression observed in this study may play a vital role in the pathogenesis of sarcoidosis. In particular, the observed significant up-regulation of the miRNAs involved in angiogenesis (miR-192, miR-221, miR-222) or EMT control (miR-192, miR-221) may indicate the presence of a significant relationship between the two processes, and may suggest that they may play a role in the pathogenesis of the disease. The obtained results highlight distinct patterns of miRNA expression in BALF cells and PB lymphocytes in sarcoidosis patients, indicating the importance of the lung microenvironment in the modulation of their expression. However, further studies are needed on larger groups of patients to develop our findings concerning these selected miRNAs and gain a greater understanding of their importance as clinical markers for disease monitoring and making therapeutic decisions.

## Additional files

Additional file 1:Figure S1. Box and whisker plots, presenting a) miR-16 b) miR-20a c) miR-27b d) miR-128a e) miR-130a f) miR-222 g) miR-let7f expression mean values in BALF cells of patients and controls. (JPEG 3074 kb) Additional file 2:Figure S2. Box and whisker plots, presenting a) miR-16 b) miR-20a c) miR-128a expression mean values in PB lymphocytes of patients and controls. (JPEG 1357 kb) Additional file 3:Figure S3. Box and whisker plots representing the expression of a) miR-15b b) miR-16 c) d) miR-27b, miR-20a e) miR128 f) miR-130a g) miR-192 h) miR-221 i) miR-222 j) miR-let7f  in BALF cells in patients in radiological stags I *vs. *II-IV (JPEG 4235 kb) Additional file 4:Figure S4. Box and whisker plots representing the expression of a) miR-15b b) miR-16 c) miR-20a d) miR-27b e) miR128 f) miR-130a g) miR-192 h) miR-221 i) miR-222 j) miR-let7f  in BALF cells in patients with the acute* vs. *insidious form of disease. (JPEG 4285 kb) Additional file 5:Figure S5. Box and whisker plots representing the expression of a) miR-15b b) miR-16 c) miR-20a d) miR-27b e) miR128 f) miR-130a  g) miR-192 h) miR-221 i) miR-221 j) miR-let7f  in BALF cells in patients with normal *vs.*abnormal spirometry (JPEG 4306 kb) Additional file 6:Figure S6. Box and whisker plots representing the expression of a) miR-15b b) miR-27b c) miR-128a  d) miR-130 e) miR-192 f) miR-221 g) miR-222 h) miR-let7f  in BALF cells in subgroup of patients with a restrictive spirometric pattern *vs. *normal spirometry (JPEG 3712 kb) Additional file 7:Figure S7. Box and whisker plots representing the expression of a) miR-15b b) miR-16  c) miR-20a d) miR-27b e) miR-128a f) miR-130a g) miR-192 h) miR-221 i) miR-222 j) miR-let7f in PB lymphocytes in patients in radiological stages I *vs.* II-IV (JPEG 4059 kb) Additional file 8:Figure S8. Box and whisker plots representing the expression of a) miR-15b b) miR-16 c) miR-20a d) miR-27b e) miR-128a f) miR-130a  g) miR-192 h) miR-221 i) miR-222 j) miR-let7f in PB lymphocytes in patients with acute *vs.* insidious form of disease. (JPEG 4053 kb) Additional file 9:Figure S9. Box and whisker plots representing the expression of a) miR-15b b) miR-16 c) miR-20a d) miR-27b e) miR128 f) miR-130a  g) miR-192 h) miR-221 i miR-222 j) miR-let7f  in PB lymphocytes in patients with obturation *vs.* abnormal spirometry. (JPEG 4159 kb)

## Abbreviations

BAL: Bronchoalveolar lavage
BALF: BAL fluid
DLCO: Diffusion capacity for carbon monoxide
EBUS-TBNA: Endobronchial ultrasonography guided transbronchial needle aspiration
FEV1: Forced expiratory volume in 1st second of expiration
FVC: Forced vital capacity
LFT: Lung function test
miRNA: microRNA
